# Supplementary material for: FAK Executes Anti-Senescence via Regulating EZH2 Signaling in Non-Small Cell Lung Cancer Cells
Source: Biomedicines. 2022 Aug 10;10(8):1937. doi: 10.3390/biomedicines10081937 (PMC9406208; doi:10.3390/biomedicines10081937)
Supplement: Supplementary file 1 [file biomedicines-10-01937-s001.zip › biomedicines-1819973-supplementary.pdf]

Supplementary Material

FAK Executes Anti-Senescence via Regulating EZH2 Signaling in Non-Small Cell Lung Cancer Cells

Hsiang-Hao Chuang [1,†], Ming-Shyan Huang [2,†], Yen-Yi Zhen [3], Cheng-Hao Chuang [1], Ying-Ray Lee [4], Michael Hsiao [5,*] and Chih-Jen Yang [1,6,7,*]

* Correspondence: mhsiao@gate.sinica.edu.tw (M.H.); chjeya@cc.kmu.edu.tw (C.-J.Y.)

1. Supplementary Figures and Table

1.1 Supplementary Table

Supplementary Table S1

KEY RESOURCES TABLE

RAGENT or RESOURCE	SOURCE	IDENTIFIER	WORKING STATUS
**Antibodies**			
Anti-beta-Actin	SIGMA	Cat# SI-A5441-.2 ml	5k dilution
Anti-EZH2	Cell Signaling Technology	Cat# 5246	1k dilution
Anti-FAK (D-1)	Santa Cruz	Cat# sc-271126	500 dilution
Anti-GFP	GeneTex	Cat# GTX113617	10k dilution
Anti-Histone 3	Proteintech	Cat# 17168-1-AP	2k dilution
Anti-Histone 3 (tri methyl K9)	Abcam	Cat# ab8898	1k dilution
Anti-Histone 3 (tri methyl K27)	Cell Signaling Technology	Cat# 9733	1k dilution
Anti-lamin A/C	GeneTex	Cat# GTX101127	1k dilution
Anti-p16	Abcam	Cat# ab108349	1k dilution
Anti-p21	Cell Signaling Technology	Cat# 2947	1k dilution
Anti-p27	GeneTex	Cat# GTX100446	1k dilution
Anti-p53	Cell Signaling Technology	Cat# 2527	1k dilution
Anti-Phospho-FAK (Tyr397)	Cell Signaling Technology	Cat# 3823	500 dilution
Anti-Phospho-FAK (Tyr576/Tyr577)	Cell Signaling Technology	Cat# 3281	500 dilution
**Cell lines**			
A549	ATCC	ATCC® CCL-185	RPMI 1640 + 10%FBS
H1299	ATCC	ATCC® CRL-5803	RPMI 1640 + 10%FBS
**Chemicals, Enzymes and Materials**			
Dulbecco's Modified Eagle Medium	Gibco	Cat# 12100-061	
Fetal bovine serum	Gibco	Cat# 10437-028	
Lipofectamine 2000	Invitrogen	Cat# 11668019	
PF-573228	MedChemExpress	Cat# HY-10461	
PhosSTOP™	Roche	ROC-04906845001	
RIPA Lysis Buffer, 10X	Millipore	Cat# 20-188	
RPMI medium1640	Gibco	Cat# 31800-089	
X-Gal Stock Solution	Millipore	Cat# BG-3-G	
WM-1119	MedChemExpress	Cat# HY-102058	
**Recombinant DNA**			
AcGFP1-C1	addgene	Plasmid #54607	
AcGFP1-FAK	In the present study, cloning PTK2 cDNA from pDONR223-PTK2 with KpnI/BamHI restriction sites onto AcGFP1-C1	N/A	

pcDNA3.1(+) EZH2-myc-His	Dr. Long-Yuan Li at NCHU	N/A
pcDNA3.1(+)/myc-His A	Invitrogen	Cat# V80020
pCMVdeltaR8.91	RNAi Core Facility, Academia Sinica	
pMDG	RNAi Core Facility, Academia Sinica	
Luciferase shRNA	RNAi Core Facility, Academia Sinica	
FAK shRNA #1	RNAi Core Facility, Academia Sinica	TRCN0000121207
FAK shRNA #2	RNAi Core Facility, Academia Sinica	TRCN0000121318
EZH2 shRNA #1	RNAi Core Facility, Academia Sinica	TRCN0000040073
EZH2 shRNA #2	RNAi Core Facility, Academia Sinica	TRCN0000040076
**Other**		
Leica DMI6000 B microscope	Leica	N/A
Nikon ECLIPSE Ti microscope	Nikon	N/A
Panoramic MIDI digital scanner	3DHISTECH	N/A

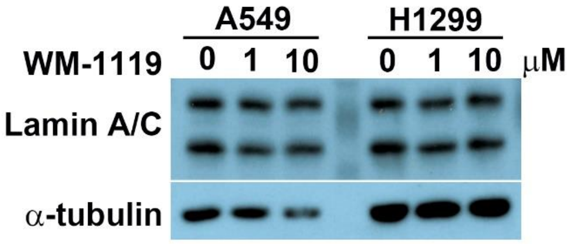

Figure S1. WM-1119 treatment does not downregulate lamin A/C expression in A549 and H1299 cells. A549 and H1299 cells were exposed to indicated concentration of WM-1119 for 48 hours, followed by lysate harvesting. It was subjected to immunoblotting for the indicated proteins. As a loading control, an anti-α-tubulin antibody was used.

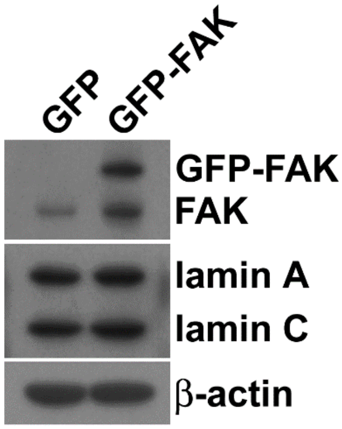

Figure S2. FAK overexpression does not increase lamin A/C expression in H1299 cells. H1299 cells were exposed to 10 μM PF-573228 for the indicated time, and then the lysates were harvested. It was subjected to immunoblotting against the indicated proteins. As a loading control, an anti-β-actin antibody was used.

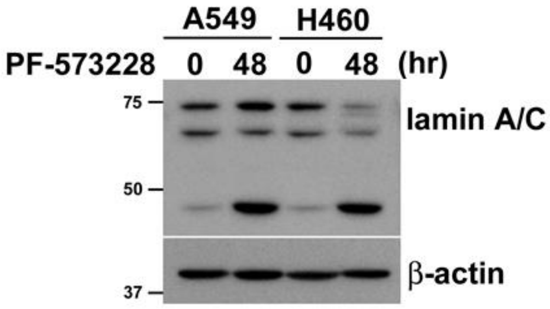

Figure S3. PF-573228 treatment induces lamin A/C cleavage. A549 or H460 cells were exposed to 10 μM PF-573228 for the indicated time, and then the lysates were harvested. It was subjected to immunoblotting against the indicated proteins. As a loading control, an anti-β-actin antibody was used. The result was showed that PF-573228 treatment partially downregulated lamin A/C through the protein cleavage pathway.
